# Outcomes for circulatory death and brainstem death pancreas transplantation with or without use of normothermic regional perfusion

**DOI:** 10.1093/bjs/znab212

**Published:** 2021-06-22

**Authors:** J A Richards, J L Roberts, A Fedotovs, S Paul, S Cottee, G Defries, C J E Watson, G J Pettigrew

**Affiliations:** 1 University of Cambridge Department of Surgery, Addenbrooke’s Hospital, Cambridge, and the NIHR Blood and Transplant Research Unit (BTRU), University of Cambridge in Collaboration with Newcastle University and in Partnership with NHS Blood and Transplant (NHSBT), UK; 2 The National Institute of Health Research (NIHR) Cambridge Biomedical Research Centre, Cambridge, UK

## Abstract

Simultaneous pancreas and kidney transplantation is the optimum treatment for patients with type 1 diabetes and renal failure, providing survival benefit over deceased donor kidney transplant alone. Here the authors demonstrate that utilization of donation after circulatory death pancreases is a safe approach to expanding the donor pool with equivalent results to donation after brainstem death transplantation. They also demonstrate that pancreas transplantation after normothermic regional perfusion is feasible, but it will require ongoing prospective study to ensure that the benefits seen for liver transplantation do not come at the expense of pancreas transplant outcomes.

## Introduction

Simultaneous pancreas and kidney transplantation is the optimal treatment for patients with type 1 diabetes and renal failure, providing survival benefit over deceased kidney transplant alone, and improved quality of life^1^^,^^2^. Waiting list mortality is compounded by a shortage of donor organs and high discard rates^3^^,^^4^. To address this, donation after circulatory death (DCD) donors have been increasingly used and now account for about 30 per cent of all simultaneous pancreas and kidney transplantations in the UK. Marked variation in the utilization of DCD pancreases exists^3^^,^^4^, which may reflect a perception that DCD grafts are ‘high risk’ compared to organs procured from brainstem dead donors due to additional warm ischaemia. Other factors include differences in withdrawal of life support and variations in the legality of antemortem interventions^5^. The authors’ early experience was similar to that of others^6^^,^^7^, in that there was no difference in short-term survival between those receiving grafts from donation after brainstem death (DBD) or conventional DCD (sDCD) donors.

Normothermic regional perfusion is a promising technique to reduce the additional ischaemic insult associated with DCD by placing the donor on a modified extracorporeal membrane oxygenator circuit in order to restore circulation of oxygenated blood to the organs following cardiorespiratory arrest. In liver transplantation, normothermic regional perfusion leads to superior outcomes compared with sDCD^8–10^. It is unclear if the benefits of normothermic regional perfusion extend to DCD pancreas transplantation.

The aim of this study was to evaluate a decade of a DCD pancreas transplant programme and a cohort of DCD pancreas transplants performed with or without normothermic regional perfusion.

## Methods

All consecutive simultaneous pancreas and kidney transplantations performed at Addenbrooke’s Hospital, Cambridge, UK from 1 August 2008 to 31 July 2018 were included in this study. Full methodology is provided in detail in the supplementary material.

## Results

A total of 211 patients (139 DBD and 72 DCD, of which 59 were sDCD and 13 normothermic regional perfusion) were included. The donor, recipient and transplant characteristics are summarized in *Table S1*.

### Patient and allograft survival

**Fig. 1:**
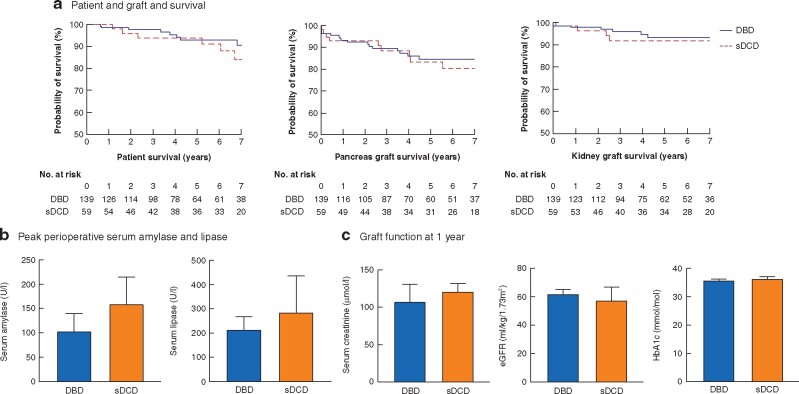
Outcomes of simultaneous pancreas and kidney transplantation from conventional donation after circulatory death compared with donation after brainstem death. **a** Kaplan–Meier plots of unadjusted patient and death censored graft survival. There was no significant difference in patient (*P* = 0.754), pancreas (*P* = 0.876) or kidney graft survival (*P* = 0.628) between those recipients receiving grafts from donation after brainstem death (DBD), conventional donation after circulatory death (sDCD) or normothermic regional perfusion donors (Mantel–Cox tests). **b** Graph of median (with 95 per cent confidence intervals) peak serum amylase and lipase levels measured in days 0–3; levels of amylase and lipase were significantly lower in DBD compared with sDCD (*P* = 0.050 and *P* = 0.040 respectively; Mann–Whitney *U* tests). **c** Graphs of median (with 95 per cent confidence intervals) serum creatinine, estimated glomerular filtration rate (eGFR) and glycated haemoglobin (HbA1c). There was no significant difference between DBD and sDCD cohorts in terms of serum creatinine (*P* = 0.085), eGFR (*P* = 0.252) or HbA1c (*P* = 0.585) at 1 year (Mann–Whitney *U* tests).

Patient survival at 1, 3, 5 and 10 years was 99.0, 96.6, 93.4 and 84.3 per cent respectively, with no significant difference between those receiving DBD or sDCD grafts (*Fig. 1a*). Death-censored pancreas and kidney graft survival at 5 years was 83.9 and 93.2 per cent respectively, with no significant difference between sDCD and DBD cohorts (*Fig. 1a*).

Delayed graft function (DGF) occurred in 33.3 per cent of renal grafts and 3.5 per cent of pancreatic grafts (*Table S2*). The rate of renal, but not pancreatic, DGF was significantly higher in the sDCD compared with the DBD cohort (*Table S3*). Serum levels of pancreatic enzymes were significantly lower in days 0–3 in the DBD compared with the DCD cohort (*Fig. 1b*). There was no significant difference in the serum creatinine, estimated glomerular filtration rate (eGFR) or glycated haemoglobin (HbA1c) at 1 year between groups (*Fig. 1c*). Rates of graft losses, thrombosis, length of stay, reoperation and episodes of rejection are included in the supplementary results (*Fig. S1*, *Tables S3* and *S4*).

### Outcomes of the normothermic regional perfusion cohort

There was no significant difference in patient or graft survival between sDCD or normothermic regional perfusion donors (*Fig. S2a*), nor in the rates of primary non-function, DGF, thrombosis, episodes of acute rejection, reoperation or readmission between sDCD or normothermic regional perfusion cohorts (*Table S4*). Peak serum lipase, but not amylase, levels were significantly lower in patients receiving normothermic regional perfusion organs compared with sDCD (*Fig. S2b*). There was no significant difference between sDCD and normothermic regional perfusion cohorts in terms of serum creatinine, eGFR or HbA1c at 1 year (*Fig. S2c*).

## Discussion

In this series of DCD simultaneous pancreas and kidney transplantation, long-term follow-up data demonstrate that patient and graft survival are equivalent for sDCD and DBD organs with no difference in graft function at 1 year. Utilization of DCD pancreases is a safe approach to expanding the donor pool with equivalent results to DBD transplantation. Also, pancreas transplantation after normothermic regional perfusion is feasible, but requires on-going prospective study to ensure that the benefits seen for liver transplantation do not come at the expense of pancreas transplant outcomes.

All outcome data for sDCD and DBD simultaneous pancreas and kidney transplantation were similar in the current series, other than the incidence of kidney DGF, which was higher for patients receiving an sDCD simultaneous pancreas and kidney transplantation (26.6 per cent *versus* 49.2 per cent; *Table S3*). This mirrors the UK rate of 49 per cent seen with isolated DCD renal transplantation^11^. sDCD transplantation was not associated with increased graft loss, major ureteric complications, rejection episodes or poorer kidney graft function at 1 year (*Table S3*).

While appropriate selection of donors and minimizing cold ischaemia time underpins successful DCD outcomes^12^, the authors think it unlikely that the comparable results achieved for DBD and DCD organs is attributable to stringent selection criteria for DCD organs – Cambridge has the lowest rate of declining DCD pancreases of any UK centre^3^ and the median Pancreas Donor Risk Index is representative of previous UK^13^, Eurotransplant^14^ and US^4^ data. Of the 45 normothermic regional perfusion donors under 50 years of age, almost half resulted in a pancreas transplant. Furthermore, although it is standard practice to abandon DCD pancreas retrieval if the donor has not reached asystole within an hour from withdrawal of life-supporting treatment, in the current series 12.2 per cent of sDCD pancreases were retrieved from donors with withdrawal of life-supporting treatment of more than 100 minutes, with the longest more than 400 minutes. Patient and graft outcomes were not different in this cohort (data not shown), in accord with previous findings for isolated kidney transplants with a prolonged agonal phase^15^.

Others have noted higher rates of graft thrombosis in DCD pancreas transplantation^16^, but this was not observed in the present series. Most episodes were incidental findings on CT (86.8 per cent) and treated non-operatively with systemic anticoagulation alone (73.7 per cent). Only 4.5 per cent of patients required operative intervention and this did not differ significantly between DBD and sDCD cohorts (Supplementary information). This fits with previous work demonstrating that most thrombi can be managed successfully with systemic anticoagulation^17^.

The present study represents a large experience of pancreas transplantations following normothermic regional perfusion. Although a small cohort, this experience nevertheless accounts for about 70 per cent of the current UK experience. The findings indicate that pancreas transplantation following normothermic regional perfusion is both feasible and offers comparable outcomes. Others have previously reported improved renal outcomes in recipients of normothermic regional perfusion compared with sDCD grafts^9^^,^^18^, but whether this is also seen in the setting of simultaneous pancreas and kidney transplantation will only become evident as experience accrues.

Lower levels of both amylase and lipase were seen in recipients of grafts from DBD compared with sDCD donors (*Fig. 1b*). Serum lipase, but not amylase, levels were also significantly lower in the normothermic regional perfusion cohort compared with sDCD (*Fig. S2b*), which may suggest less severe graft pancreatitis^19^. This warrants further study to confirm or refute this observation.

Given the waiting list mortality and known survival benefits of simultaneous pancreas and kidney transplantation compared with renal transplant alone for diabetic patients^1^^,^^12^, it is difficult to justify the large discrepancies in utilization of DCD pancreases^3^^,^^4^. As with other organs, this may have resulted from a cognitive bias, whereby a single poor outcome has disproportionately influenced the perception of the risks associated with DCD transplantation^20^.

## Supplementary Material

znab212_Supplementary_Information
