# Supplementary material for: Outcomes for circulatory death and brainstem death pancreas transplantation with or without use of normothermic regional perfusion
Source: Br J Surg. 2021 Jun 22;108(12):1406–8. doi: 10.1093/bjs/znab212 (PMC10364865; doi:10.1093/bjs/znab212)
Supplement: znab212_Supplementary_Information [file znab212_supplementary_information.docx]

**Methods**

All consecutive primary pancreas transplantations performed at Addenbrooke’s Hospital, Cambridge from 1^st^ August 2008 to 31^st^ July 2018 were included in this study. Follow up was collected until August 2019.

**Study design and setting**

Simultaneous pancreas and kidney transplants from both DBD and controlled DCD donors (Maastricht category 3) were included in this study. Since April 2010, organ retrieval in the UK has been performed by a dedicated National Organ Retrieval Service (NORS), according to an agreed protocol (1).

*Recipient Selection & Pancreas Allocation*

In the UK, type 1 diabetic patients can be wait-listed for SPK transplantation provided they are receiving dialysis or have an estimated or measured Glomerular Filtration Rate (GFR) of 20mls/min or less (2). Insulin dependent type 2 diabetic patients are also eligible if they have a calculated BMI of less than 30 kg/m^2^.

Before December 2010, pancreas allocation was undertaken regionally and recipients were selected by the on-call transplant team on the basis of blood group, HLA mismatch, presence of donor‐specific HLA antibodies, waiting time and donor age. Since December 2010, all pancreases in the UK, whether being considered for solid organ or islet cell transplantation, must be offered through the National Pancreas Allocation Scheme (NPAS) (3). Recipients are chosen based on an algorithm, scoring recipients on their HLA antibody calculated reaction frequency (similar to calculated panel reactive antibodies), donor-recipient age difference, donor BMI, requirement for dialysis, waiting time and distance between the donor and implanting hospitals.

*Donor Selection*

Only DBD and controlled DCD donors fulfilling NHSBT guidelines were considered as potential donors (4). The only pancreas-specific absolute contraindications were diabetes mellitus (excluding insulin requirements whilst on the intensive care unit), acute necrotizing pancreatitis, chronic pancreatitis, pancreatic malignancy, donor BMI >40kg/m^2^, donors <15kg, DBD donors ≥ 66 years and DCD donors aged ≥56 years (4). Donor offers were considered on an individual basis but relative contraindications to pancreas transplantation included: history of recent pancreatic trauma; significant hyperamylasaemia / hyperglycaemia; high insulin requirements; or donor BMI >30 kg/m^2^.

*Standard DCD (sDCD) pancreas retrieval*

WLST typically occurred in the intensive care unit or anaesthetic room. Agonal phase is defined as the time from withdrawal of life supporting treatment to circulatory arrest, and during this time vital signs are continuously monitored. After circulatory arrest, an independent medical practitioner confirmed death after a 5-minute observation time and only after this period could procurement begin (5). UK organ retrieval teams wait a minimum of three hours from WLST before abandoning retrieval; we have previously demonstrated that it is possible to pursue donation for longer (6). In the UK, intravenous administration of heparin or other pre-mortem interventions aimed specifically at facilitating organ donation are not permissible.

A rapid procurement technique via a midline laparotomy is utilised, with dual aortic and portal venous perfusion with University of Wisconsin solution (Belzer UW, Bridge to Life Ltd., Columbia, USA) containing 25,000 units of heparin per litre in the first two litres with additional topical cooling with crushed frozen saline. The liver-pancreas block is then either split *in situ* or on the backtable according to the preference of the retrieving surgeon. Our DCD pancreas transplant programme started in August 2008.

*Normothermic Regional Perfusion*

We have previously described our NRP circuit in detail (7, 8) and aim for target flows for isolated abdominal NRP of 2.5‐3 L/min and 4‐6 L/min for thoracoabdominal NRP. Briefly, abdominal NRP is established via cannulae in the aorta or right common iliac artery plus the inferior vena cava or right common iliac vein, with a cross-clamp occluding the descending thoracic aorta. Thoracoabdominal NRP was performed via cannulae in the ascending aorta and IVC (either directly or via the right atrial appendage), or cannulating the abdominal aorta and IVC, with a cross-clamp placed across the origins of the brachiocephalic trunk, left common carotid and left subclavian arteries. The aortic arch is vented to ensure no cerebral flow (9). Given ante‐mortem cannulation and heparinisation are not permitted in the UK, we add 50 000 units heparin to the NRP circuit.

Typically, we perfuse the abdominal organs for 2 hours to allow viability testing of the liver with limited mobilisation of the abdominal organs prior to *in situ* perfusion with cold University of Wisconsin preservation solution. The liver-pancreas block is then either split *in situ* or on the backtable according to the preference of the retrieving surgeon. The NRP programme in our centre started in September 2010, with the first pancreas transplanted following NRP in April 2013.

*DBD pancreas retrieval*

In patients satisfying UK brainstem death criteria, full mobilisation of the organs is performed during the warm phase, prior to systemic heparinisation and aortic cross-clamping, with the immediate commencement of dual aortic and portal venous perfusion with University of Wisconsin solution and additional topical cooling with crushed frozen saline. The pancreas block is then retrieved having been typically dissected free from the liver *in situ* (or occasionally on the backtable according to the preference of the retrieving surgeon).

**Implant technique**

*Pancreas Transplantation*

All pancreas transplants were performed intraperitoneally using a standard technique with arterial inflow from the common or external iliac artery using a donor iliac arterial “Y” conduit, portal drainage to the IVC and enteric exocrine drainage via a Roux-en-Y duodenojejunostomy, as we have previously described (10).

*Kidney Transplantation*

The donor kidney is subsequently implanted in an extraperitoneal pouch in the left iliac fossa accessed via the existing midline incision. Vessels are anastomosed to the respective common or external iliac vessels and ureterovesical anastomosis performed to the dome of the recipient bladder over a double J stent. All patients were admitted to a level 2 bed for at least the first 24 hours post-transplant.

**Peri- & post-operative management**

*Immunosuppression*

All patients received subcutaneous alemtuzumab at induction, with tacrolimus plus mycophenolate maintenance and no steroids.

*Anti‐thrombotic and anti-microbial prophylaxis*

Daily subcutaneous low molecular weight heparin was administered from day 1 until discharge and oral aspirin (75 mg daily) was started at discharge.

All recipients received 5 days IV antibiotic prophylaxis (2008-2015: meropenem, 2015 onwards: piperacillin/tazobactam) and anti-candidal prophylaxis with fluconazole for 7 days.

Cytomegalovirus (CMV) seropositive recipients received oral valganciclovir for 3 months, and CMV seronegative recipients of organs from CMV seropositive donors received oral valganciclovir for 6 months. CMV seronegative recipients of organs from CMV seronegative donors were given aciclovir prophylaxis against herpes simplex and varicella zoster for 3 months.

**Study definitions**

The transplant outcomes used in the study are defined in Supplemental Table 1. Patient survival was unadjusted and graft survival was censored for death.

Cold ischemia time (CIT) was defined as the time from commencement of *in situ* cold perfusion until reperfusion in the recipient. Asystolic warm ischemia time was defined as the time from circulatory arrest to the start of cold perfusion or commencement of NRP (as appropriate) and the agonal warm ischemia or withdrawal time as the time from WLST to circulatory arrest.

Pancreas Donor Risk Index (PDRI) was calculated according to the original description by Axelrod and colleagues (11). Estimated glomerular filtration rate (eGFR) was used to determine renal function and was calculated with the Modification of Diet in Renal Disease formula (12).

**Statistical analysis**

Statistical methods are specifically referred to in the results section. Given the sample size, the analysis plan was to compare the outcomes of sDCD against the “gold standard” of DBD, and then the pilot NRP data comparing outcomes of patients receiving DCD allografts: i.e. NRP against sDCD. Briefly, categorical transplant characteristics were compared using chi-squared and continuous by Mann-Whitney test. Unadjusted graft and patient survival were calculated using Kaplan-Meier plots. Analysis was performed using Prism 8 for Mac OSX (Graphpad Software, La Jolla, USA).

**Ethical approval**

This study was given approval by Cambridge University Hospitals NHS Foundation Trust Clinical Audit Department (PRN3056). It was deemed that additional ethical approval was unnecessary.

**SUPPLEMENTARY RESULTS**

There were no significant demographic differences between recipients of DBD, sDCD and NRP grafts (Supplemental Table 2).

Two (1.0%, 2 DBD) pancreas and two (1.0%, 2 DBD) kidney grafts suffered primary non-function (PNF), with two (1.0%, 1 sDCD, 1 DBD) pancreas grafts removed intra-operatively due to bleeding from the portal vein, but the incidence did not differ between those receiving grafts from DBD or sDCD donors (Table 1).

One patient died secondary to mesenteric infarction within the first year. 5/198 (2.5%) renal grafts did not survive 1 year (2 failures to thrombosis, 2 to rejection, 1 death (bowel ischemia)). In the remaining 193 (97.5%) grafts, the median serum creatinine was 111 μmol/L (range 50-258) and eGFR was 61 ml/min/1.73m^2^ (range 20-168).

Of the pancreas grafts, 16/198 (8.1%) did not survive 1 year (2 removed intra-operatively, 10 failures from thrombosis, 3 rejection & 1 death). The median HbA1c at one-year post-transplant was 36mmol/mol (range 20-61).

*Thrombosis*

Overall, 9/198 (4.5%) patients had significant pancreatic graft thrombosis (defined as requiring thrombectomy or graft pancreatectomy if intraoperatively deemed not salvageable). Including those identified incidentally on imaging, 17.7% (35/198) patients were identified as having a thrombus within the graft. Thirty (85.7%) were detected on CT scanning, one (2.9%) on USS prompting immediate return to theatre, one (2.9%) was identified at the index transplant operation and three (8.6%) at re-operation due to clinical suspicion of thrombosis. Twenty-six (74.2%) of identified thrombi were managed by anticoagulation alone. There was no difference between the rates of thrombosis, early thrombosis or need for operative management of a thrombotic event between those receiving a DBD or sDCD graft (Table 1).

*Length of stay, readmission and reoperations*

The median total length of hospital stay for recipients was not significantly different between groups (DBD 15 days, sDCD 17 days, Mann-Whitney p=0.259). Furthermore, there was no difference in the requirement for ICU (level 3) admission post-transplant between patients who received DBD or sDCD grafts during their index admission (Table 1). However, sDCD graft recipients were significantly more likely to require readmission to a level 2 bed during their index admission (13.7% DBD versus 28.8% sDCD, Table 1).

Following discharge from hospital, 48.0% were readmitted (all cause admission excluding elective day procedure) within the first 90 days and 66.2% within the first year following transplantation (Table 1). There was no difference in the 90-day or 1-year readmission rate between groups (Table 1). The overall re-operation rate was significantly higher in the DCD (40.7%) compared to DBD cohort (25.9%, Table1). Return to theatre for the management of post-operative bleeding accounted for 20/60 (33.3%), but there was no significant difference in the rate of return for haemorrhage between cohorts (DBD: 11/36 (30.6%), DCD: 9/24 (37.5%), Mann-Whitney p=0.590). Major ureteric complications occurred in 5.1% patients, but the incidence did not differ according to donor type (Table 1).

*Rejection*

Overall, 33/198 (16.7%) of recipients had one or more episodes of rejection within the first year and 42/198 (21.2%) during the study period; this was not determined by the type of donation (Supplemental Table 2). There was no significant difference between the number of mismatches in HLA-A, -B and -DR and episodes of rejection in the first year or throughout the study period (Supplementary Figure 1).

**Supplementary Figures**

**Supplementary Figure 1: Rejection and HLA-A, -B and –DR mismatches**

**Supplementary Figure 2: Outcomes of SPK from sDCD compared to NRP**

Kaplan-Meier plots of unadjusted patient and death censored graft survival were plotted (**A**). There was no significant difference in patient (Mantel-Cox p=0.451), pancreas (p=0.514) or kidney graft survival (p=0.429) between those recipients receiving grafts from sDCD or NRP donors.

Graph of median (+/- 95% confidence interval) peak serum amylase and lipase levels measured in days 0-3 were plotted (**B**); levels of lipase (Mann-Whitney p=0.008), but not amylase (p=0.175), were significantly lower in patients receiving NRP organs compared to sDCD.

Graphs of median (+/- 95% confidence interval) of serum creatinine, eGFR and HbA1c were plotted (**C**). There was no significant difference between sDCD and NRP cohorts in terms of serum creatinine (Mann-Whitney p=0.515), eGFR at 1 year (p=0.212) or HbA1c (p=0.495).

**SUPPLEMENTARY TABLES**

**Supplementary Table 1: Donor and Recipient demographics**

Continuous variables are described as the mean with range and analysed by Kruskall-Wallis (or Mann-Whitney if only 2 groups). Categorical variables are expressed as an absolute number with percentage and analysed by Pearson’s chi-squared test.

Abbreviations: BMI: body mass index; CVA: cerebral vascular accident; HLA: Human Leukocyte Antigen; WLST: Withdrawal of Life-Supporting Treatment.

| **Variable** | **DBD** | **sDCD** | **NRP** |  |
| --- | --- | --- | --- | --- |
|  | 139 | 59 | 13 |  |
| ***Donor*** | | | | |
| **Age** (years) | 36 (7-59) | 32 (14-59) | 28 (16-48) | **p=0.030** |
| **Gender** |  |  |  | p=0.066 |
| Male | 64 (46.0%) | 33 (55.9%) | 10 (76.9%) |  |
| Female | 75 (54.0%) | 26 (44.1%) | 3 (23.1%) |  |
| **BMI** (kg/m^2^) | 24 (15-33) | 23 (16-33) | 24 (16-30) | p=0.708 |
| **Cause of death** |  |  |  | **p<0.00001** |
| CVA/ intracranial haemorrhage | 82 (59%) | 18 (30.5%) | 3 (23%) |  |
| Head trauma | 11 (7.9%) | 9 (15.3%) | 7 (54%) |  |
| Hypoxic brain injury | 33 (23.7%) | 30 (50.8%) | 2 (15%) |  |
| Brain tumour | 3 (2.2%) | 0 (0%) | 0 (0%) |  |
| Other | 10 (7.2%) | 2 (3.4%) | 1 (8%) |  |
| **Pancreas Donor Risk Index (11)** | 1.50 (0.59-2.88) | 1.72 (0.95-3.74) | 1.54 (1.07-2.62) | p=0.092 |

| ***Recipient*** | | | | |
| --- | --- | --- | --- | --- |
| **Age (years)** | 43 (24-62) | 44 (26-59) | 40 (24-52) | p=0.539 |
| **Gender** |  |  |  | p=0.131 |
| Male | 81 (58.3%) | 39 (66.1%) | 11 (84.6%) |  |
| Female | 58 (41.7%) | 20 (33.9%) | 2 (15.4%) |  |
| **BMI** (kg/m^2^) | 26 (16-36) | 26 (20-33) | 25 (21-31) | p=0.494 |
| **Diabetes** |  |  |  | p=1.000 |
| Type 1 diabetes | 138 (99.3%) | 59 (100%) | 13 (100%) |  |
| Type 2 diabetes | 1 (0.7%) | 0 (0%) | 0 (0%) |  |
| **Duration of diabetes** (years) | 28 (11-47) | 30 (9-54) | 25 (15-42) | p=0.323 |
| **Pre-transplant renal status** |  |  |  | p=0.520 |
| Pre-dialysis | 45 (32.4%) | 17 (28.8%) | 6 (46.2%) |  |
| Haemodialysis | 60 (43.2%) | 23 (39.0%) | 3 (23.1%) |  |
| Peritoneal dialysis | 34 (24.5%) | 19 (32.2%) | 4 (30.8%) |  |
| **For patients commenced on dialysis: Duration** (months) | 21 (0-186) | 19 (2-68) | 8 (2-18) | p=0.084 |
| **Total number of HLA mismatches (HLA‐A, ‐B, -DR)** | 4 (1-6) | 4 (1-6) | 4 (3-6) | p=0.676 |
| **Pancreas Cold Ischemia Time** (minutes) | 610 (231-868) | 583 (286-823) | 517 (361-692) | **p=0.0271** |
| **Kidney Cold Ischemia Time** (minutes) | 790 (403-1191) | 769 (545-1031) | 698 (550-863) | p=0.0584 |
| **Time from WLST (or Cross-clamp in DBD) to cold perfusion or NRP (as appropriate)** (minutes) | n/a | 54 (8-419) | 38 (16-185) | p=0.642 |
| **Time from Arrest (or Cross-Clamp in DBD) to start of cold perfusion or NRP (as appropriate)** (minutes) | n/a | 12 (3-22) | 13 (10-18) | p=0.180 |
| **Anastomosis time- pancreas** (minutes) | 40 (19-125) | 40 (18-84) | 45 (30-93) | p=0.235 |
| **Anastomosis time- kidney** (minutes) | 43 (10-74) | 44 (8-68) | 40 (26-53) | p=0.592 |

**Supplementary Table 2: Outcome definitions**

|  | *Definition* |
| --- | --- |
| **Delayed Graft Function** |  |
| Pancreas | Requirement for administration of exogenous insulin to control hyperglycaemia within the first 7 days post-transplantation. |
| Kidney | Requirement for within the 7 days post-transplantation, excluding dialysis for hyperkalaemia within the first 24 hours post-op. |
| **Primary Non-Function (PNF)** |  |
| Pancreas | Failure of the pancreas or kidney graft to ever function, irrespective of underlying aetiology, excluding those grafts removed intra-operatively. |
| Kidney |  |
| **Graft Loss or Failure** |  |
| Pancreas | Graft pancreatectomy or return to insulin or oral hypoglycaemic medication. |
| Kidney | Graft nephrectomy or return to renal replacement therapy. |
| **Rejection** | Episode of either pancreas or kidney rejection as defined below. |
| Pancreas | Pancreas rejection was defined either as:   - On the basis of a pancreatic biopsy using the Banff pancreas allograft criteria (13).   OR (more typically)   - Clinical / biochemical / imaging suspicion of pancreatic rejection with a contemporaneous renal biopsy suggesting rejection. |
| Kidney | Renal allograft rejection was biopsy‐proven and defined by Banff criteria (14). In the rare instance that biopsy was contraindicated (e.g. significant hypertension, coagulopathy), then the diagnosis was made on the basis of clinical and biochemical suspicion and the commencement of antirejection treatment. |
| **Graft survival** | Time in days from pancreas or kidney transplantation to graft failure (as defined above), censored to date of death with a functioning pancreas or kidney grafts respectively or date of last follow-up in those with grafts still functioning at time of analysis. |
| **Patient Survival** | Patient survival is the time from transplant to death, censored to date of last follow-up in those patients alive at time of analysis. |
| **Readmission** | All cause readmission to hospital, excluding elective day case procedures (e.g. ureteric stent removal). |
| **Graft thrombosis** | Episode of thrombosis (partial or complete) within allograft vasculature detected clinically in theatre or on imaging.  This was further subdivided as early (<90 days) and late (>90 days) as well as complete (surgically managed) or partial (managed with systemic anticoagulation). |
| **Major Ureteric Complication** | Requiring for placement of nephrostomy, cytoscopic stent placement or need for surgical ureteric reconstruction (15). |

**Supplementary Table 3: Graft function and patient outcomes of DBD compared to sDCD**

Categorical variables are expressed as an absolute number with percentage and analysed by Fisher’s exact test.

|  | **Total** | | | **DBD** | | | | **sDCD** | |  | |
| --- | --- | --- | --- | --- | --- | --- | --- | --- | --- | --- | --- |
|  | n | | % | n | % | | | n | % |  |  |
|  | 198 | |  | 139 |  | | | 59 |  |  | |
| **Primary non-function (Pancreas)** | | | | | | | | | | p=1.000 | |
|  | 2 | | (1.0%) | 2 | | | (1.4%) | 0 | (0%) |  |  |
| **Primary non-function (Kidney)** | | | | | | | | | | p=1.000 | |
|  | 2 | | (1.0%) | 2 | | | (1.4%) | 0 | (0%) |  |  |
| **Delayed Graft Function (Pancreas)** | | | | | | | | | | p=0.427 | |
|  | 7 | | (3.5%) | 4 | | | (2.9%) | 3 | (5.1%) |  |  |
| **Delayed Graft Function (Kidney)** | | | | | | | | | | **p=0.003** | |
|  | 66 | (33.3%) | | 37 | | | (26.6%) | 29 | (49.2%) |  |  |
| **Thrombotic Event Requiring Surgery (pancreatectomy / thrombectomy)** | | | | | | | | | | p=1.000 | |
|  | 9 | (4.5%) | | 6 | | | (4.3%) | 3 | (5.1%) |  |  |
| **All graft thrombosis** | | | | | | | | | | p=0.102 | |
|  | 35 | (17.7%) | | 29 | | | (20.9%) | 6 | (10.2%) |  |  |
| **Early graft thrombosis** | | | | | | | | | | p=0.128 | |
|  | 29 | (14.6%) | | 24 | | | (17.3%) | 5 | (8.5%) |  |  |
| **Episode of Acute Rejection within 1 year** | | | | | | | | | | p=0.678 | |
|  | 33 | | (16.7%) | 22 | | (15.8%) | | 11 | (18.6%) |  |  |
| **Any episode of Acute Rejection** | | | | | | | | | | p=0.851 | |
|  | 42 | (21.2%) | | 29 | | | (20.9%) | 13 | (21.2%) |  |  |
| **Required admission to ICU (level 3) bed** | | | | | | | | | | p=0.722 | |
|  | 50 | (25.2%) | | 34 | | | (24.5%) | 16 | (27.1%) |  |  |
| **Required readmission to Level 2 bed** | | | | | | | | | | **p=0.016** |  |
|  | 36 | (18.2%) | | 19 | | | (13.7%) | 17 | (28.8%) |  |  |
| **Readmission within 1^st^ 90 days** | | | | | | | | | | p=0.643 |  |
|  | 95 | (48.0%) | | 65 | | | (46.8%) | 30 | (50.8%) |  |  |
| **Readmission within 1^st^ year** | | | | | | | | | | p=0.070 |  |
|  | 131 | (66.2%) | | 86 | | | (61.9%) | 45 | (76.3%) |  |  |
| **Reoperation within 90 days** | | | | | | | | | | **p=0.044** |  |
|  | 60 | (30.3%) | | 36 | | | (25.9%) | 24 | (40.7%) |  |  |
| **Major Ureteric Complications (nephrostomy or ureteric reimplantation)** | | | | | | | | | | p=0.287 |  |
|  | 10 | (5.1%) | | 9 | | | (6.5%) | 1 | (1.7%) |  |  |

**Supplementary Table 4: Graft function and patient outcomes of SPK from DCD donors**

Categorical variables are expressed as an absolute number with percentage and analysed by Fisher’s exact test.

|  | **Total** | | | **sDCD** | | **NRP** | |  |
| --- | --- | --- | --- | --- | --- | --- | --- | --- |
|  | n | | % | n | % | n | % |  |
|  | 72 | |  | 59 |  | 13 |  |  |
| **Primary non-function (Pancreas)** | | | | | | | | p=0.181 |
|  | 1 | | (1.4%) | 0 | (0%) | 1 | (7.7%) |  |
| **Primary non-function (Kidney)** | | | | | | | | p=1.000 |
|  | 0 | | (0%) | 0 | (0%) | 0 | (0%) |  |
| **Delayed Graft Function (Pancreas)** | | | | | | | | p=1.000 |
|  | 3 | | (4.2%) | 3 | (5.1%) | 0 | (0%) |  |
| **Delayed Graft Function (Kidney)** | | | | | | | | p=0.357 |
|  | 33 | (45.8%) | | 29 | (49.2%) | 4 | (30.8%) |  |
| **All graft thrombosis** | | | | | | | | p=0.349 |
|  | 9 | (12.5%) | | 6 | (10.2%) | 3 | (23.1%) |  |
| **Early graft thrombosis** | | | | | | | | p=0.151 |
|  | 8 | (11.1%) | | 5 | (8.5%) | 3 | (23.1%) |  |
| **Thrombotic Event Requiring Surgery (pancreatectomy or thrombectomy)** | | | | | | | | p=0.558 |
|  | 4 | (5.6%) | | 3 | (5.1%) | 1 | (7.7%) |  |
| **Episode of Acute Rejection within 1 year** | | | | | | | | p=0.708 |
|  | 14 | | (19.4%) | 11 | (18.6%) | 3 | (23.1%) |  |
| **Any episode of Acute Rejection** | | | | | | | | p=1.000 |
|  | 16 | (22.2%) | | 13 | (21.2%) | 3 | (23.1%) |  |
| **Required admission to ICU (level 3) bed** | | | | | | | | p=1.000 |
|  | 19 | (26.4%) | | 16 | (27.1%) | 3 | (23.1%) |  |
| **Required readmission to Level 2 bed** | | | | | | | | p=1.000 |
|  | 21 | (29.2%) | | 17 | (28.8) | 4 | (30.8%) |  |
| **Readmission within 1^st^ 90 days** | | | | | | | | p=1.000 |
|  | 102 | (48.3%) | | 30 | (50.8%) | 7 | (53.8%) |  |
| **Readmission within 1^st^ year** | | | | | | | | p=1.000 |
|  | 55 | (76.3%) | | 45 | (76.3%) | 10 | (76.9%) |  |
| **Reoperation within 90 days** | | | | | | | | p=0.346 |
|  | 27 | (37.5%) | | 24 | (40.7%) | 3 | (23.1%) |  |
| **Major Ureteric Complications (nephrostomy or ureteric reimplantation) (15) (15)** | | | | | | | | p=1.000 |
|  | 1 | (1.4%) | | 1 | (1.7%) | 0 | (0%) |  |

**References**

1. NHS Blood and Transplant: National Standards for Organ Retrieval from Deceased Donors 2016 [Available from: <https://nhsbtdbe.blob.core.windows.net/umbraco-assets-corp/1405/nors_retrieval_standards.pdf>.

2. NHSBT. POL185/6: Pancreas Transplantation- Patient Selection 2019 [Available from: <https://nhsbtdbe.blob.core.windows.net/umbraco-assets-corp/17326/pancreas-selection-policy-pol185.pdf>.

3. NHSBT. POL199/10: Pancreas Transplantation: Organ Allocation 2019 [Available from: <https://nhsbtdbe.blob.core.windows.net/umbraco-assets-corp/17479/pancreas-allocation-policy-pol199.pdf>.

4. NHSBT. POL188/6: Clinical contraindications to approaching families for possible organ donation 2018 [Available from: <https://nhsbtdbe.blob.core.windows.net/umbraco-assets-corp/6455/contraindications_to_organ_donation.pdf>.

5. Academy of Medical Royal Colleges: A code of practice for the diagnosis and confirmation of death 2008 [Available from: <http://aomrc.org.uk/wp-content/uploads/2016/04/Code_Practice_Confirmation_Diagnosis_Death_1008-4.pdf>.

6. Reid AWN, Harper S, Jackson CH, Wells AC, Summers DM, Gjorgjimajkoska O, et al. Expansion of the Kidney Donor Pool by Using Cardiac Death Donors with Prolonged Time to Cardiorespiratory Arrest. American Journal of Transplantation. 2011;11(5):995-1005.

7. Watson CJE, Hunt F, Messer S, Currie I, Large S, Sutherland A, et al. In situ normothermic perfusion of livers in controlled circulatory death donation may prevent ischemic cholangiopathy and improve graft survival. American Journal of Transplantation. 2018.

8. Butler AJ, Randle LV, Watson CJ. Normothermic regional perfusion for donation after circulatory death without prior heparinization. Transplantation. 2014;97(12):1272-8.

9. Manara A, Shemie SD, Large S, Healey A, Baker A, Badiwala M, et al. Maintaining the permanence principle for death during in situ normothermic regional perfusion for donation after circulatory death organ recovery: A United Kingdom and Canadian proposal. American Journal of Transplantation. 2020;20(8):2017-25.

10. Amin I, Butler AJ, Defries G, Russell NK, Harper SJF, Jah A, et al. A single-centre experience of Roux-en-Y enteric drainage for pancreas transplantation. Transplant International. 2017;30(4):410-9.

11. Axelrod DA, Sung RS, Meyer KH, Wolfe RA, Kaufman DB. Systematic Evaluation of Pancreas Allograft Quality, Outcomes and Geographic Variation in Utilization. American Journal of Transplantation. 2010;10(4):837-45.

12. Levey AS. A More Accurate Method To Estimate Glomerular Filtration Rate from Serum Creatinine: A New Prediction Equation. Annals of Internal Medicine. 1999;130(6).

13. Drachenberg CB, Odorico J, Demetris AJ, Arend L, Bajema IM, Bruijn JA, et al. Banff Schema for Grading Pancreas Allograft Rejection: Working Proposal by a Multi-Disciplinary International Consensus Panel. American Journal of Transplantation. 2008;8(6):1237-49.

14. Solez K, Colvin RB, Racusen LC, Haas M, Sis B, Mengel M, et al. Banff 07 Classification of Renal Allograft Pathology: Updates and Future Directions. American Journal of Transplantation. 2008;8(4):753-60.

15. Richards JA, Jones K, Moinuddin Z, Pettigrew GJ. Response to: Prophylactic Ureteric Stents in Renal Transplant Recipients: A Multicenter Randomized Controlled Trial of Early Versus Late Removal. American Journal of Transplantation. 2017;17(11):2990-.
